# *In vitro* tetraploid induction and generation of tetraploids from mixoploids in *Dioscorea zingiberensis*

**DOI:** 10.4103/0973-1296.59966

**Published:** 2010-02-13

**Authors:** He-Ping Huang, Shan-Lin Gao, Lan-Lan Chen, Kun-Hua Wei

**Affiliations:** 1 *Department of Genetics and Breeding, China Pharmaceutical University, Nanjing, 211198, R.P. China*; 2 *Department of School of Pharmacy, Anhui University of Traditional Chinese Medicine, Hefei 230031, R.P. China*; 3 *Department of Anhui Key Laboratory of Modernized Chinese Material, Hefei 230031, R.P, China*

**Keywords:** Callus, colchicine, *Dioscorea zingiberensis*, leaf characteristic, polyploidy

## Abstract

This article describes an efficient colchicine-mediated technique for the *in vitro* induction of tetraploids in *Dioscorea zingiberensis* and its confirmation by flow cytometry. Buds immersed in 0.2% colchicine solution for 36 hours prior to culture induced as high as 35.6% tetraploid plants. Colchicine-induced tetraploids remained stable after six months in soil. Leaf characteristics of diploids and tetraploids in *D. zingiberensis* were compared. It was determined that the leaf sizes of glasshouse-grown plants and stomatal sizes of both *in vitro* and glasshouse-grown plants were suitable parameters for identifying putative tetraploids in *D. zingiberensis*. Besides generating tetraploids, this technique generated mixoploids in *D. zingiberensis*. Calli derived from mixoploid leaves were induced to form buds and shoots. Individual shoots were classed as diploid, mixoploid, and tetraploid by flow cytometry. This callus-based technique could be employed when a genome-doubling agent generated mixoploids, but no tetraploids.

## INTRODUCTION

Diosgenin is a steroidal compound, important in the pharmaceutical industry as a natural source of steroidal hormones.[1–2] In China, *Dioscorea zingiberensis* is the dominant resource for the production of diosgenin. On the other hand, the rhizomes of *D. zingiberensis* have the curative effects of relieving cough and pain, in detoxification, and in reducing swelling. Culturing superior varieties of *D. zingiberensis* is important in order to improve the content and yield of diosgenin. Polyploid plants have been used in plant breeding to develop superior varieties.[3–5]

There have been several *in vitro* studies on *D. zingiberensis*.[6–9] However, reports on *in vitro* polyploidy induction in *D. zingiberensis* are limited.[10–11] We sought a protocol to regenerate polyploidy plants via bud clusters from seed explants of *D. zingiberensis* by colchicine treatments. The technique of *in vitro* polyploid induction with colchicine has been employed in many plants, such as banana, mulberry, and corn.[12–14] However, only a few cases of polyploid medicinal plants have been reported.[15] Tetraploid plants of *Datura stramonium* have one-to-two times higher alkaloid content in leaves, stems, and roots as compared to diploids.[15] The content of alkaloid in tetraploid plants of *Atropa belladonna* is 154% of that in the diploid plant.[16] The ultimate aim of this study is to develop pure tetraploids for use in the field, as the leaves, roots, and rhizomes of tetraploid plants are usually bigger than those of diploid plants.

Although *in vitro* induction of polyploidy permitted mass production of pure tetraploids, it could also generate mixoploids (i.e., chimeras consisting of diploid and tetraploid cells). Rapidly growing root tips of diploid plants were selected for counting chromosome numbers. Diploid cells with chromosome numbers 2n = 20 were determined and no aneuploid plant was reported. Consequently, an effective method was required to identify tetraploids. Flow cytometry analysis of nuclear DNA content is being increasingly used for high-throughput ploidy screening.[17] The leaf characteristics of diploid and tetraploid plants are compared, to determine whether any markers can be used to identify putative tetraploids in this species. Furthermore, this article describes a callus-based technique that permits the generation of tetraploid *D. zingiberensis*, using mixoploid *D. zingiberensis* as the starting material.

## MATERIALS AND METHODS

### Plant material

Seeds of *D. zingiberensis* (2X = 20) were obtained from the Wudang Mountain area, Hubei province, China. The original plant was identified by the Department of Genetics and Breeding at the China Pharmaceutical University.

### Seed disinfection, germination, and *in vitro* multiplication

The seeds of *D. zingiberensis* were sterilized by immersion in 2% v/v sodium hypochlorite solution (containing 3-5 drops/L of Tween-20) for eight minutes followed by immersion in 0.1% w/v mercuric chloride solution for five minutes. The seeds were then rinsed with sterile distilled water three to five times and then transferred to a Petri dish containing sterile filter paper, to remove the excess surface water. The surface-sterilized seeds were placed into a Murashige and Skoog (MS)[18] semi-solid culture medium. The inoculated seeds were incubated in an illuminated chamber under a 16-hour photoperiod of 1200 lux light intensity at 25°C, to initiate germination. After 15 days, the seedlings were transferred to a shoot multiplication medium: An MS medium supplemented with 0.8 mg/l BA and 0.2 mg/l NAA.

### Tetraploid induction

Liquid MS medium supplemented with 2% dimethyl sulfoxide (DMSO) and filter-sterilized colchicine (final colchicine concentrations: 0.1, 0.2, and 0.3%) was used for tetraploid induction. The experimental plant material was bud clusters (0.5-1 cm) excised from *in vitro* grown cultures. The buds were immersed in MS liquid medium containing the respective concentrations of colchicine described earlier as well as in colchicine-free MS medium and incubated at 25°C on an orbital shaker (100 rpm) for 12, 24, 36, 48, or 60 hours. A total of 30 explants were used per treatment. Buds were washed thrice (2-3 minutes) with sterile distilled water and transferred to the shoot multiplication medium.

### Establishment of tetraploid plants

All of the tetraploid buds placed on the rooting medium (solid MS medium with 1/2-macronutrient concentration, supplemented with 0.5 mg/l NAA) formed roots. Rooted plant were placed in a glasshouse and kept at 90-100% relative humidity for the first week. All tetraploids, either maintained *in vitro* for eight cycles of culture or grown in the glasshouse for 6 months, were stable with no sign of ploidy reversion or chimerism.

### Flow cytometry analysis of ploidy level

Ploidy of treated plants was measured by flow cytometry (Becton Dickinson Immunocytometry Systems). Flow cytometer measurements were taken according to the protocol of Arumuganathan and Earle,[19] modified by Dikson *et al*.[20] with double filtration according to Bukhari.[21] The samples were centrifuged for 5 seconds, which was enough to convert the small nuclei into pellets.

Relative fluorescence intensities of at least 10,000 particles were measured. Nuclei from diploid *D. zingiberensis* shoots were used as a standard. Measurements were taken as soon as the shoots had grown sufficiently for taking a 25 mg sample.

### Estimation of leaf characteristics

Leaf characteristics were obtained from 30-day-old *in vitro* material about 1 cm^2^ in size, and from six-month-old fully established glasshouse plants about 5 cm^2^ in size. For the stomatal measurements, an area of about 0.1 cm^2^ on the upper epidermis of the leaves was smeared with PVA (polyvinyl acetate wood glue). After the glue dried, the PVA impression was removed using a strip of cellotape (transparent adhesive cellulose tape). The tape was then stuck on to a microscope slide. Two leaves were chosen from the same part of each of five diploid control plants and each of five tetraploid plants. Twenty stomata were measured for each leaf.

### Callus generation

Leaf explants (0.1 cm^2^) from *in vitro*-grown mixoploids were cultured on MS medium containing different concentrations of cytokinin (BA) and auxin (NAA, 2, 4-D). Calli were proliferated on MS media supplemented with BA (0.5-1.0 mg/l), NAA (0-0.5 mg/l), and 2, 4-D (1.0-2.0 mg/l). To generate buds, the calli were cultured on MS medium supplemented with BA (0-2.0 mg/l) and NAA (0-0.5 mg/l) for a month and then the buds were maintained on shoot multiplication medium. The ploidy level of calli-derived shoots was evaluated by flow cytometry. The shoots were selected from each mixoploid, but data analysis was not performed, as the aim was to determine whether the tetraploid shoots could be retrieved from the mixoploid callus. All data in this study were statistically analyzed using the Duncan's multiple range test.

## RESULTS

### Survival and growth of colchicine-treated buds

The effects of colchicine on the growth of explants was assessed a month after treatment [Table 1]. Non-growing, brown buds were considered to be dead. All the explants in the colchicine-free treatment (data not shown) and in the 12 hour, low-colchicine (0.1%) treatments survived [Table 1]. However, each of the other treatments showed some lethality, with all the 0.3% colchicine treatments having greater than 70% lethality.

**Table 1:** Effect of different concentrations and treatment duration of colchicine on polyploidy induction (Mean ± standard error) in Dioscorea zingiberensis (all data are in percentages)

Colchicine (%)	Characteristics	Time (h)				
		
		12	24	36	48	60
0.1	Diploid	76.2 ± 11.6	8.7 ± 2.1	0.0 ± 0.0	0.0 ± 0.0	0.0 ± 0.0
	Mixoploid	23.8 ± 5.9	71.2 ± 15.6	72.7 ± 12.8	59.3 ± 8.2	64.6 ± 6.8
	Tetraploid	0.0 ± 0.0^a^	15.5 ± 3.8^b^	22.8 ± 5.6^c^	13.9 ± 2.1^b^	0.0 ± 0.0^a^
	Dead	0.0 ± 0.0	4.6 ± 1.7	4.5 ± 1.2	26.8 ± 3.8	35.4 ± 4.9
0.2	Diploid	0.0 ± 0.0	0.0 ± 0.0	0.0 ± 0.0	0.0 ± 0.0	0.0 ± 0.0
	Mixoploid	41.8 ± 9.3	40.2 ± 7.8	14.6 ± 2.9	34.0 ± 3.3	16.1 ± 2.6
	Tetraploid	24.2 ± 5.7^c^	24.0 ± 5.6^c^	35.6 ± 6.6^c^	22.3 ± 5.2^c^	15.1 ± 2.8^b^
	Dead	34.0 ± 7.1	35.8 ± 5.3	49.8 ± 10.1	43.7 ± 5.9	68.8 ± 6.9
0.3	Diploid	0.0 ± 0.0	0.0 ± 0.0	0.0 ± 0.0	0.0 ± 0.0	0.0 ± 0.0
	Mixoploid	18.6 ± 4.1	20.9 ± 4.0	18.7 ± 3.6	16.9 ± 4.3	17.4 ± 5.0
	Tetraploid	11.2 ± 2.4^b^	7.4 ± 1.3^b^	7.2 ± 1.5^b^	0.0 ± 0.0^a^	0.0 ± 0.0^a^
	Dead	70.2 ± 7.9	71.7 ± 9.5	74.1 ± 8.9	83.1 ± 5.7	82.6 ± 7.7

With each tetraploid row, means followed by the same letter are not significantly different at the P = 0.05 level, by Duncan's multiple range test; means followed by a, b, and c are significantly different at the *P* = 0.05 level each other

Another visible effect of colchicine was the delayed growth of treated explants. The initiation of bud growth occurred within three-to-four days in untreated explants and 10-15 days in colchicine-treated explants. After a month of growth, all colchicine-treated explants had significantly shorter shoots than untreated explants.

### Flow cytometry analysis of colchicine-treated explants

**Figure 1:**
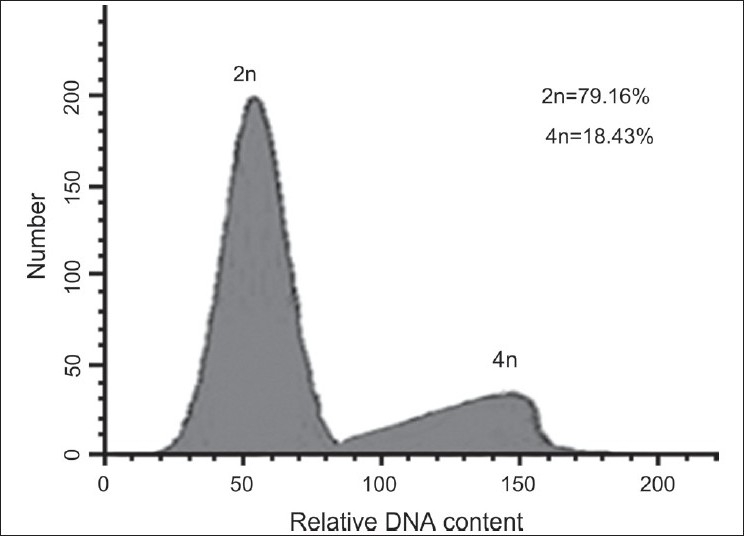
DNA-histograms of nuclei isolated from shoots of a diploid

**Figure 2:**
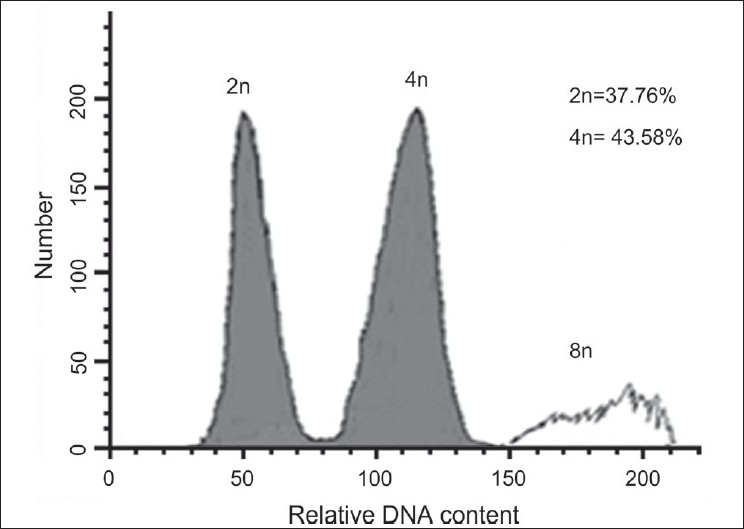
DNA-histograms of nuclei isolated from shoots of a mixoploid

**Figure 3:**
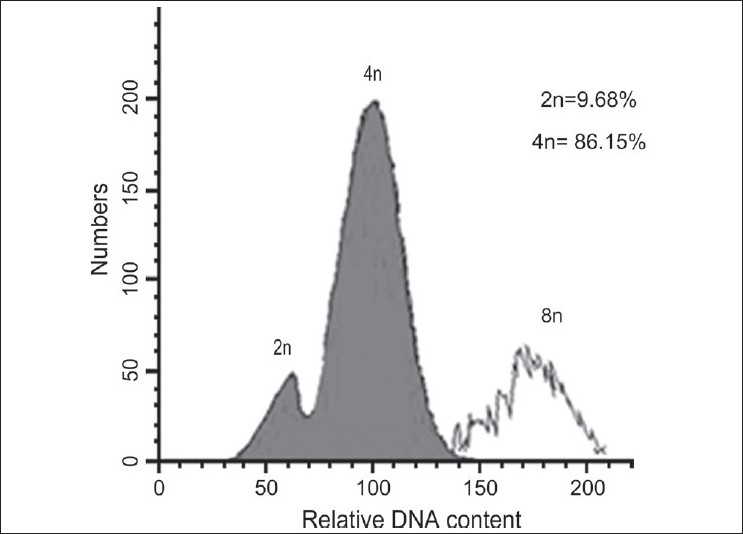
DNA-histograms of nuclei isolated from shoots of a tetraploid

All colchicine-treated explants that grew were subjected to flow cytometry to determine their ploidy. Individual explants were classified as diploid, mixoploid, and tetraploid according to the peaks obtained by flow cytometry [Table 1]. Representative examples are shown in Figures 1–3. Diploids possessed a small percentage of nuclei with a tetraploid complement of DNA [Figure 1], which represented nuclei in the late S or G2 phase of the cell cycle. Similarly, mixoploid and tetraploid explants possessed a small percentage of octaploid nuclei [Figures 2–3]. The plants with approximately equal numbers of diploid and tetraploid nuclei were regarded as mixoploid plants. A number of explants generated a polyploidy response, as mixoploids and tetraploids were obtained [Table 1].

### Morphological differences between diploid and tetraploid *D. zingiberensis*

**Figures 4 and 5:**
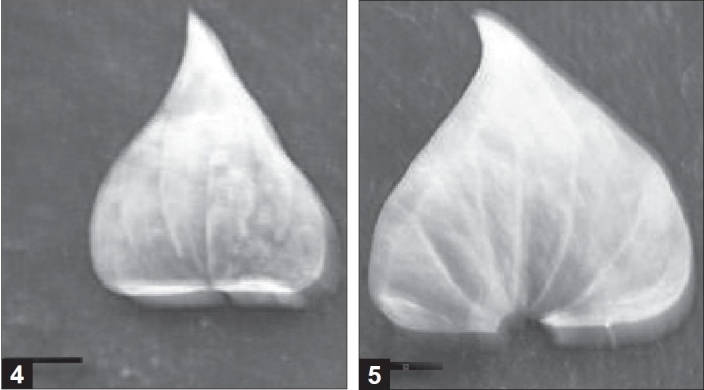
Leaves of diploid and tetraploid plants in *Dioscorea zingiberensis* from glasshouse

In most cases, the leaves of tetraploid plants appeared normal in shape as compared to the diploid plants. The length and width of 30-day-old diploid and tetraploid *in vitro* leaves were not significantly different, but those of the six-month-old, glasshouse-grown plants did differ significantly [Table 2, Figures 4 and 5]. These characteristics were significantly different when the same ploidy material from *in vitro* and glasshouse-grown leaves were compared [Table 2].

**Table 2:** Leaf characteristics (mean ± standard error) of diploid and tetraploid *Dioscorea zingiberensis*

Characteristics	Diploid (*in vitro*)	Tetraploid (*in vitro*)	Diploid (glasshouse)	Tetraploid (glasshouse)
Leaf length (mm)	11.3 ± 1.5^a^	11.8 ± 1.6^a^	21.2 ± 1.8^b^	32.3 ± 2.4^c^
Leaf width (mm)	11.0 ± 0.9^a^	11.4 ± 1.1^a^	20.4 ± 1.5^b^	31.7 ± 1.5^c^
Stomatal length (μm)	11.7 ± 1.3^a^	19.9 ± 1.8^b^	21.1 ± 2.3^b^	29.2 ± 1.7^c^
Stomatal width (μm)	8.8 ± 1.1^a^	18.1 ± 1.9^b^	19.2 ± 2.0^b^	27.6 ± 1.9^c^

with each row, means followed by the same letter are not significantly different at P = 0.05 Level, by Duncan's multiple range test

Each leaf was obtained from the same part of diploid [Figure 4] and tetraploid [Figure 5] plant grown in the glasshouse (bar: 0.75 cm).

**Figures 6 and 7:**
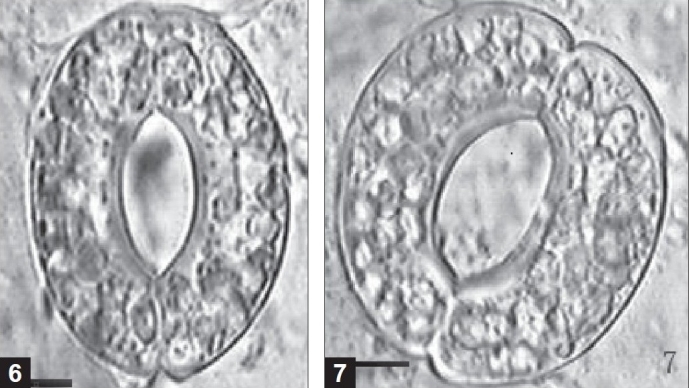
Stomata of diploid and tetraploid plants in *Dioscorea zingiberensis* from glasshouse

Each stoma was obtained from the same part of diploid [Figure 6] and tetraploid [Figure 7] leaves in the glasshouse (bar: 6.25 × 10^−4^ cm). *In vitro* diploid and tetraploid leaves as well as glasshouse-grown diploid and tetraploid leaves were measured and found to be significantly different [Table 2, Figures 6 and 7]. Overall, the tetraploids possessed longer and wider stomata. There was also a significant difference when the same ploidy material was grown under different conditions [Table 2]. In both diploids and tetraploids, the glasshouse- grown material possessed longer and wider stomata.

### Establishment of callus culture from mixoploids

**Figures 8 and 9:**
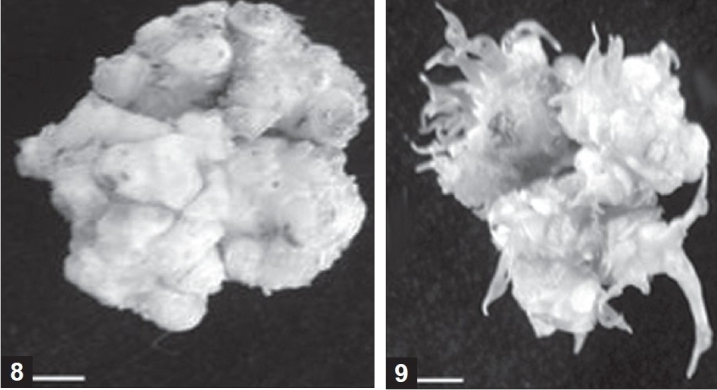
Leaf calli elicited from a mixoploid plant in *Dioscorea zingiberensis*

Callus was not observed when only a cytokinin or an auxin was present in the medium (data not shown), however, when they were used in combination, all treatments promoted callus growth [Table 3]. The combination of 1.0 mg/l BA, 0.2 mg/l NAA, and 2.0 mg/l 2, 4-D elicited the highest response (96.8%) of callus formation [Table 3, Figure 8].

**Table 3:** Effect of plant growth regulators on the percentage of mixoploid leaf explants callusing (mean ± standard error) in *Dioscorea zingiberensis* (data scored after a month and obtained from 30 explants per treatment)

Cytokinin (mg/l) BA	Auxin (mg/l)		Calli formation (%)

	NAA	2,4-D
0.5	0.0	2.0	72.9 ± 2.1^b^
0.5	0.2	0.0	50.2 ± 1.9^a^
0.5	0.5	1.0	85.1 ± 3.0^c^
1.0	0.0	1.0	47.8 ± 2.7^a^
1.0	0.2	2.0	96.8 ± 4.1^d^
1.0	0.5	0.0	85.6 ± 3.7^c^

Means followed by a, b, c, and d are significantly different at the *P* = 0.05 level each other, by Duncan's multiple range test

Callus elicited from a mixoploid plant a month after the leaf explant was placed in the medium [Figure 8] and shoot buds generated from the leaf callus of mixoploid plant [Figure 9] (bar: 1.00 cm).

### Initiation of shoot buds from callus

The best response for shoot bud formations in callus cultures was elicited with 2.0 mg/l BA, 0.2 mg/l NAA [Table 4]. This treatment was significantly different from the other treatments and caused 84.7% of the calli to generate shoot buds, with approximately seven shoot buds per callus [Table 4, Figure 9]. After a month, calli with shoot buds were placed on fresh media containing the same combinations of BA and NAA for shoot formation. Callus-derived shoots of mixoploids were again analyzed by flow cytometry. Tetraploid shoots were obtained from all mixoploids with the average percentage (17.3%).

**Table 4:** Regeneration of shoot buds (mean ± standard error) from calli of mixoploid *Dioscorea zingiberensis* (data scored after a month and obtainedfrom 30 calli per treatment)

BA (mg/l)	NAA (mg/l)	Percentage of calli with shoot buds	Buds per callus
0.0	0.0	0.0 ± 0.0^a^	0.0 ± 0.0^a^
1.0	0.2	27.2 ± 2.3^b^	2.1 ± 0.3^b^
1.0	0.5	50.4 ± 4.1^c^	5.3 ± 0.7^c^
2.0	0.2	84.7 ± 6.9^e^	6.9 ± 1.2^d^
2.0	0.5	68.1 ± 5.2^d^	5.4 ± 0.9^c^

Means followed by a, b, c, d and e are significantly different at the P 5=0.05 level each other, by Duncan's multiple range test

## DISCUSSIONS

The inverse relationship between colchicine concentration and explant survival was expected and is in agreement with the *ex vitro* studies using other plant types.[22,23]

At the same time, colchicine caused a slow growth of treated explants, which may be due to a physiological disturbance, resulting in a reduced rate of cell division.[24] On the other hand, both untreated and colchicine-treated explants grew equally well in a subculture, suggesting that colchicine only caused an initial retardation of growth, as observed in the *ex vitro* studies.[23]

In most cases, all the explants were affected by colchicine treatment. The exceptions were 0.1% colchicine (12 hours, 24 hours) treatments, where some diploids remained unaffected [Table 1]. The highest percentage of tetraploid induction occurred in the 0.2% colchicine 36-hour treatment (35.6%). However, the 0.1% colchicine (36 hours) and 0.2% colchicine (12, 24, and 48 hours) treatment also generated a substantial number of tetraploids (>20%) and were not significantly different from the 0.2% colchicine 36-hour treatment [Table 1].

Furthermore, the morphological features of polyploid plants were evaluated to determine whether they could be used to identify putative tetraploids. On the basis of our results, the leaf sizes of glasshouse-grown plants and stomatal sizes of both *in vitro* and glasshouse-grown plants were useful parameters for identifying putative tetraploids in *D. zingiberensis*. The utility of stomatal size in distinguishing plants with different ploidy levels was used in other plant types.[12–13,17,23] As a number of mixoploids had been identified [Table 1], we considered it possible to obtaintetraploids from these mixoploids by generating shoots from the tetraploid cells of the leaf callus. From our experiment, it was clear that tetraploid *D. zingiberensis* plants could be generated from mixoploids via the callus. Generally, in this study, an efficient procedure for tetraploid *D. zingiberensis* generation has been demonstrated. DNA measurement via flow cytometry makes it possible to quickly evaluate hundreds of putative tetraploids. The tetraploid plants grown in the glasshouse have larger leaves and stomata than the diploid plants. These tetraploid plants promise to be of important genetic and breeding value and will be used for further selection.
